# Functional evaluation for adequacy of MDCK-lineage cells in influenza research

**DOI:** 10.1186/s13104-019-4134-2

**Published:** 2019-02-26

**Authors:** Hsin-Chung Tsai, Caitlin W. Lehman, Chi-Chieh Lin, Sen-Wei Tsai, Chuan-Mu Chen

**Affiliations:** 1 Department of Surgery, Taichung Hospital, Ministry of Health and Welfare, 199 San Min Rd., Sec. 1, Taichung, Taiwan; 2 Department of Life Sciences, The iEGG and Animal Biotechnology Center, National Chung Hsing University, 145 Xinda Rd., Taichung, 402 Taiwan; 3 School of System Biology, George Mason University, Manassas, VA USA; 4 Department of Biomedical Sciences, National Chung Hsing University, Taichung, Taiwan; 5 Department of Physical Medicine and Rehabilitation, Taichung Tzu Chi Hospital, Buddhist Tzu Chi Medical Foundation, Taichung, Taiwan

**Keywords:** Influenza virus, MDCK, MCDK/London, Mv1Lu, Ribavirin

## Abstract

**Objective:**

Influenza is an acute respiratory disease caused by the influenza virus which circulates annually in populations of different species. Madin-Darby Canine Kidney (MDCK) is the most widely utilized cell-line for conducting influenza research. However, the infectivity of various influenza strains in MDCK cells is not equivalent and the productivity of viral propagation is also limited.

**Results:**

We tested the functional adequacy of two MDCK-lineage cell lines, conventional MDCK and MDCK/London, were evaluated by assessing their infectivity of different influenza viral strains with focus forming assays and the cellular toxicity caused by influenza infections by lactate dehydrogenase assay. Moreover, the sensitivity of cells in the presence of the antiviral agent ribavirin was assessed by MTT assay. Our results showed that MDCK/London cells efficiently propagate virus across all influenza viruses tested, are comparable to the utility of Mv1Lu cells, and are superior to conventional MDCK cells in replicating virus as indicated by an increase in virus of three to four logs, particularly in H3N2 infection. Also, the MDCK/London cells were more sensitive to the presence of antiviral drug than conventional MDCK cells. In conclusion, MDCK/London cell line could be a better platform for influenza studies and vaccine development.

## Introduction

Influenza virus causes pandemics and seasonal epidemics worldwide, leading to 5–15% of the population becoming infected and 50,000 deaths annually in United States [1]. Frequently complicated by bacterial infection, influenza infection can lead to influenza-associated pneumonia which, in combination, is implicated in up to 8.4% of all reported deaths to the 122 Cities Mortality Reporting System in the United States [2]. Researchers have widely utilized influenza-susceptible cell lines such as MDCK or Vero for pathogenesis investigation as well as vaccine developments, particularly MDCK culture-derived vaccine against influenza has been approved by European Medicines Agency [3, 4]. As such, it is critical to select the cell type utilized for conducting influenza research and to interpret data generated from these various cell types. Currently, several cell lines in addition to MDCK and Vero have been reported to be susceptible to influenza virus infection such as hamster lung (HmLu-1), monkey kidney (JTC-12), human colon intestinal epithelium cell line (CACO-2), and mink lung epithelial cells (Mu1Lv) [5–7].

The MDCK/London (MDCK/Ln) cell line is also a suitable substrate to grow and isolate influenza virus and is available for purchase on the website of Influenza Reagent Resource (IRR; FR-58). In 1985, MDCK/London cells were originated and developed from the Common Cold Laboratory in Salisbury, UK. MDCK/Ln cells display enhanced sensitivity to influenza infections [8, 9]. A recent study revealed that the MDCK/Ln has a faster growth rate and reflects the influenza infection more sensitively in regards to PFU, HA, and NA titers compared to MDCK/SIAT1 and conventional MDCK cells [10]. However, a thorough, in-depth, and functional comparison of MDCK/Ln and conventional MDCK cells has yet to be demonstrated. Here, we compare the viral propagation of MDCK-lineage cells with another cell line, Mv1Lu, which has been shown to be susceptible to influenza infections [6], by a high throughput focus forming assay which has been described previously [11].

## Main text

**Fig. 1:**
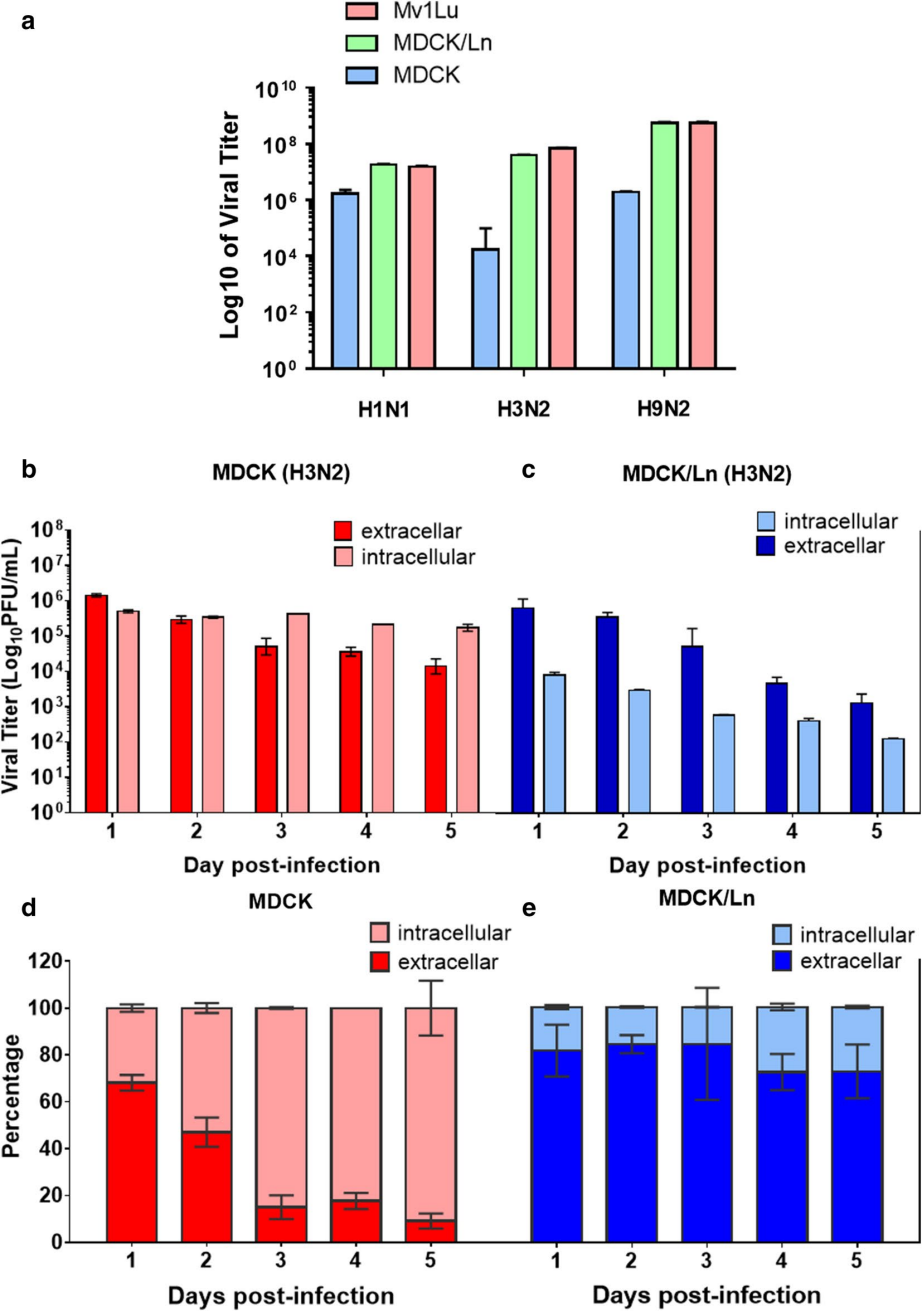
Viral yield of Influenza viruses from MDCK-lineage and Mv1Lu cells were titrated. **a** MDCK (Md), MDCK/London (Md/Ln), and Mv1Lu (Mv) cells were utilized as substrates for titration of H1N1, H3N2, and H9N2 with M.O.I. of 0.1 by focus forming assays. The PFU titers (**b**, **c**) and the percentages (**d**, **e**) of intracellular and extracellular H3N2 virus in conventional MDCK and MDCK/London cells over consecutive 5 days of infection (M.O.I. = 0.1) period. *VC* viral control. Images were presented from one of three independent experiments with similar observations

To validate the infectivity of influenza viruses propagated in egg embryos (H1N1, ATCC: VR1520; H3N2, ATCC: VR544; H9N2, ATCC: VR1642), we directly performed plaque assays to titrate these viruses in different cells, including MDCK-lineage cells and Mv1Lu cell. The results in Fig. 1a showed that the viral titers of H1N1, H3N2, and H9N2 in MDCK/Ln cells were higher than those in conventional MDCK cells and comparable to the titers in Mv1Lu cells. Among these influenza strains, H3N2 exhibited the most divergent outcomes in conventional MDCK and MDCK/Ln cells. As such, we selected the H3N2 strain to investigate the productivity of influenza virions in MDCK and MDCK/Ln cells by measuring intracellular and extracellular viral titers over time. In order to assess intracellular and extracellular viral titers, supernatant from both cell lines infected with H3N2 was collected and the infected cells were subsequently sonicated to release intracellular virions followed by titration via plaque assays. As illustrated in Fig. 1b–e, the intracellular viral titers in MDCK cells increased over time, reaching over 80% of total virions by day 3 post-infection but there was less than 20% of total viral titers were released from conventional MDCK cells (Fig. 1b, d), On the contrary, the extracellular viral titers in MDCK/Ln cells were at the range of 70–80% of total virions and the intracellular titers were much lower than extracellular titers (20–30%) (Fig. 1c, e), suggesting that influenza virions could be more readily released from MDCK/Ln cells which may due to more efficient viral package and contributed to the higher titers outside host cells in MDCK/Ln, but not MDCK cells.

**Fig. 2:**
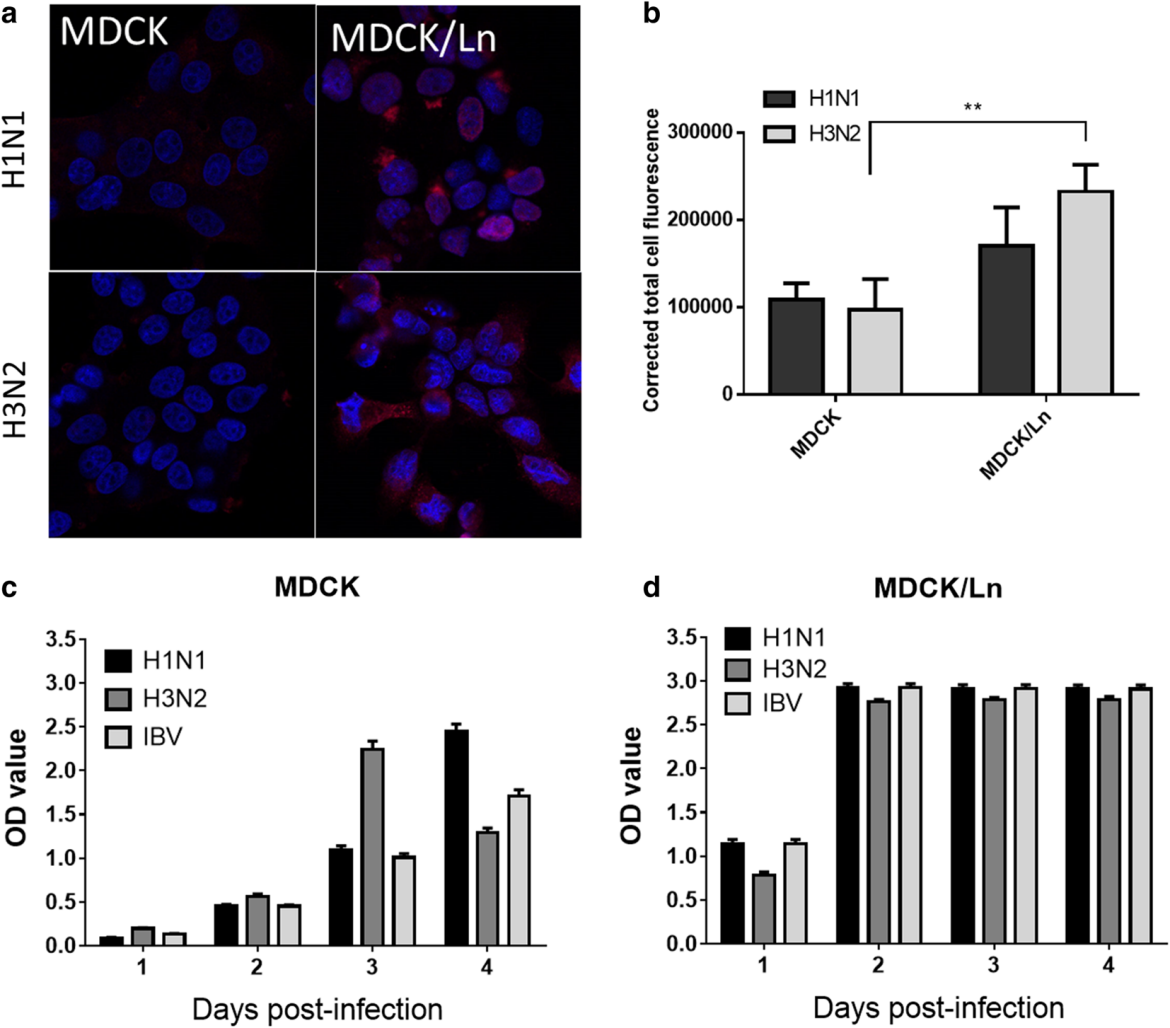
The comparison of influenza infectivity in MDCK and MDCK/London cell lines. **a** Cells were infected with H1N1 (upper panel) and H3N2 (lower panel) for 48 h.p.i. followed by fixation and stained with anti-flu NP antibody after BSA blocking. Images were taken by confocal microscopy in a ×680 magnification. Red fluorescence indicates the location of influenza viruses while blue fluorescence stained by DAPI shows the nucleus of the cell. **b** The corrected total cell fluorescence (CTCF) for H1N1 or H3N2 was calculated by ImageJ software v.1.8.0. ** indicates the p value is less than 0.01. The cytotoxicity of **c** conventional MDCK and **d** MDCK/London cells were determined by the concentrations of LDH in culture medium after influenza infections (M.O.I. of 0.1) at indicated time points, measured by LDH assays. The OD values proportionally correspond to the LDH concentration in accordance with the manufacturer’s instructions

Next, we observed the H1N1- and H3N2-infected MDCK and MDCK/Ln cells by confocal microscopy at 48 h-post infection (h.p.i.), visualizing the influenza nuclear protein (NP) with anti-H1N1 (MBS: PAB7123P) and anti-H3N2 (MBS: PAB7124P) antibodies to further examine the infectivity of conventional MDCK and MDCK/Ln cells. As seen in Fig. 2a, the confocal images revealed increased intracellular fluorescence of H1N1 and H3N2 viral proteins in MDCK/Ln cells compared to conventional MDCK cells which was confirmed by corrected total cell fluorescence (CTCF) quantification of the fluorescence intensity of NP in both cell types with ImageJ software (Fig. 2b) [12]. However, there is no such significance also determined by Student’s t test between H1N1 in both cell lines, and these data to some degree corresponded with our previous findings in Fig. 1b, c.

Next, we performed functional assays in order to further characterize MDCK/Ln cells as a model for influenza infection. We evaluated the cytotoxicity resulting from influenza infections by Lactate dehydrogenase (LDH) assays (CytoTox 96^®^, Promega). The cytotoxicity, as measured by the increase of LDH, in both MDCK and MDCK/Ln cells continually increased following infection with H1N1, H3N2, and influenza B virus (IBV; ATCC: VR1883). However, compared to the gradual increase of LDH concentration in conventional MDCK cells, the LDH concentration in MDCK/Ln cells increased robustly by Day 2 post-infection and remained at higher levels (Fig. 2c, d).

**Fig. 3:**
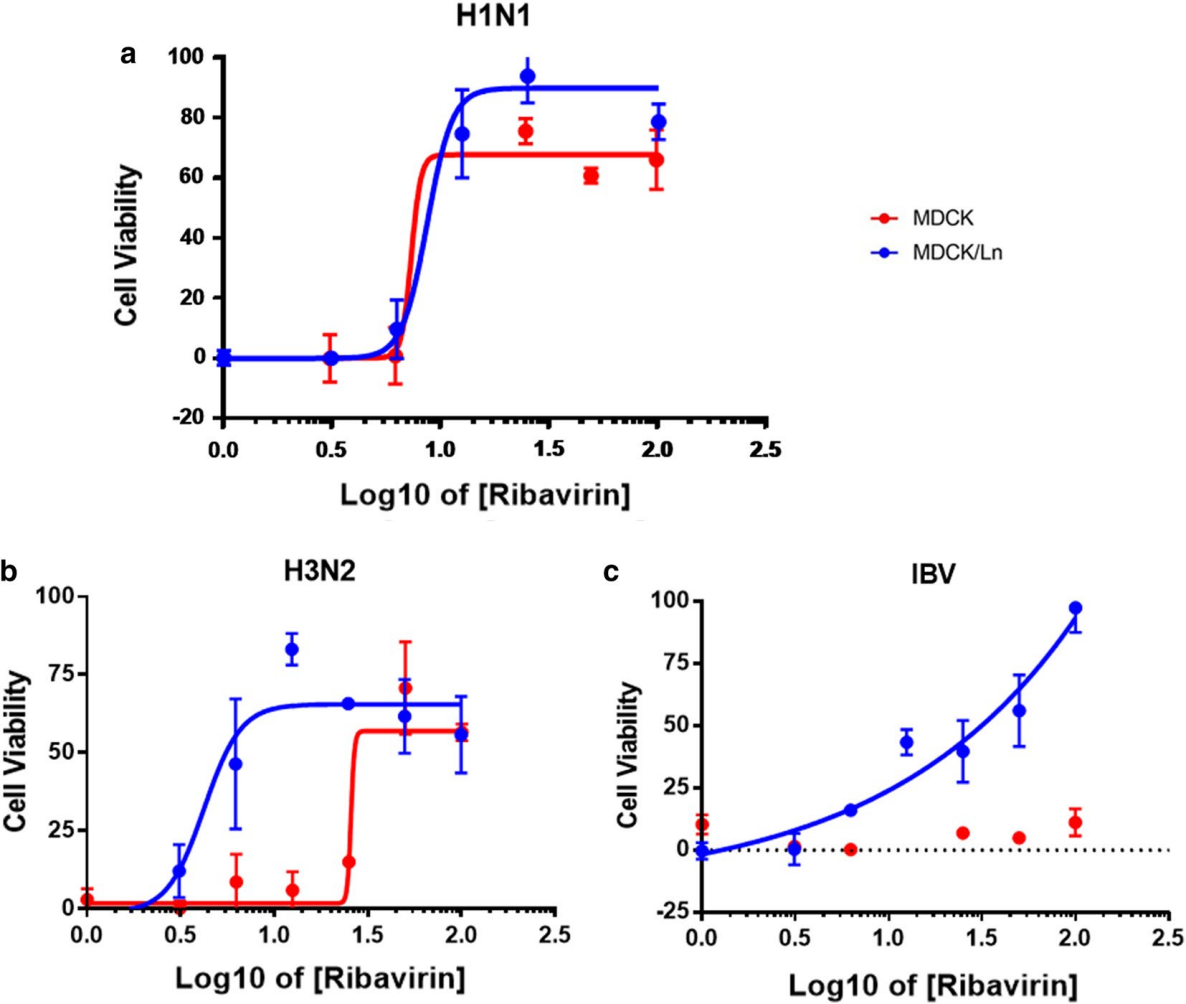
Efficacy of ribavirin in MDCK and MDCK/London cells. MDCK and MDCK-London cells were infected by **a** H1N1, **b** H3N2, and **c** influenza B viruses at M.O.I. of 0.1 followed by treatment with different concentrations of ribavirin for 72 h. Cell viability was determined by MTT assay and quantified by a colorimetric ELISA reader at wavelength 540 nm. The 50% of effective concentration (EC50) of ribavirin were calculated based on the cell viability results

Lastly, we investigated the sensitivity of MDCK-lineage cells in response to an antiviral compound. The broad spectrum and FDA-approved antiviral agent ribavirin (1-beta-d-ribofuranosyl-1,2,4-triazole, Sigma-Aldrich) [13] was utilized to test whether its antiviral efficacy can be reliably reflected in these MDCK-lineage cell lines. We infected the cells with H1N1, H3N2, or IBV at multiplicity of infection (M.O.I.) of 0.1 along with different concentrations of ribavirin, washed out the viral inoculum and ribavirin with PBS and finally added fresh complete culture medium containing fresh ribavirin for the next 72 h of incubation. The antiviral efficacy was defined as cell viability at end point measured by MTT (3-(4,5-dimethylthiazol-2-yl)-2,5-diphenyltetrazolium bromide) tetrazolium reduction assay [14]. The results presented in Fig. 3 suggest that the MDCK/Ln cells exhibit enhanced sensitivity to the ribavirin treatments than conventional MDCK cells in response to these three influenza strains. The 50% of effective concentration (EC50) against H1N1, H3N2, and IBV in MDCK/Ln were 8.623, 4.249, and 27.548 μM, respectively. Conversely, except for the H1N1 infection, the EC50 in conventional MDCK cells were higher (Fig. 3b) or unable to be determined (Fig. 3c). Our findings align with previous observations of differences in the EC50 of ribavirin in H1N1 and H3N2 strains [10, 15, 16]. Importantly, our results demonstrate that MDCK/Ln cells could be a more suitable cell line than for determining antiviral efficacy against influenza infections.

## Limitations

MDCK cells are widely used in influenza research and vaccine development [17, 18], but the viral yield and efficacy of MDCK-derived vaccines are limited [19, 20]. While further investigation of the underlying mechanisms is required, our functional evaluation for MDCK-lineage cells as a model for influenza infections provide an alternative aspect of MDCK/Ln cells. We believe that our research findings regarding MDCK/Ln cells can be used to further the flu vaccine development and influenza-related research.
